# Agronomic Characteristics Related to Grain Yield and Nutrient Use Efficiency for Wheat Production in China

**DOI:** 10.1371/journal.pone.0162802

**Published:** 2016-09-15

**Authors:** Limin Chuan, Ping He, Tongke Zhao, Huaiguo Zheng, Xinpeng Xu

**Affiliations:** 1 Institute of Information on Science and Technology of Agriculture, Beijing Academy of Agriculture and Forestry Sciences, Beijing, P.R. China; 2 Ministry of Agriculture Key Laboratory of Crop Nutrition and Fertilization, Institute of Agricultural Resources and Regional Planning, Chinese Academy of Agricultural Sciences, Beijing, P.R. China; 3 International Plant Nutrition Institute China Program, Beijing, P.R. China; 4 Institute of Plant Nutrition and Resources, Beijing Academy of Agriculture and Forestry Sciences, Beijing, P.R. China; Huazhong University of Science and Technology, CHINA

## Abstract

In order to make clear the recent status and trend of wheat (*Triticum aestivum* L.) production in China, datasets from multiple field experiments and published literature were collected to study the agronomic characteristics related to grain yield, fertilizer application and nutrient use efficiency from the year 2000 to 2011. The results showed that the mean grain yield of wheat in 2000–2011 was 5950 kg/ha, while the N, P_2_O_5_ and K_2_O application rates were 172, 102 and 91 kg/ha on average, respectively. The decrease in N and P_2_O_5_ and increase in K_2_O balanced the nutrient supply and was the main reason for yield increase. The partial factor productivity (PFP, kg grain yield produced per unit of N, P_2_O_5_ or K_2_O applied) values of N (PFP-N), P (PFP-P) and K (PFP-K) were in the ranges of 29.5~39.6, 43.4~74.9 and 44.1~76.5 kg/kg, respectively. While PFP-N showed no significant changes from 2000 to 2010, both PFP-P and PFP-K showed an increased trend over this period. The mean agronomic efficiency (AE, kg grain yield increased per unit of N, P_2_O_5_ or K_2_O applied) values of N (AEN), P (AEP) and K (AEK) were 9.4, 10.2 and 6.5 kg/kg, respectively. The AE values demonstrated marked inter-annual fluctuations, with the amplitude of fluctuation for AEN greater than those for AEP and AEK. The mean fertilizer recovery efficiency (RE, the fraction of nutrient uptake in aboveground plant dry matter to the nutrient of fertilizer application) values of N, P and K in the aboveground biomass were 33.1%, 24.3% and 28.4%, respectively. It was also revealed that different wheat ecological regions differ greatly in wheat productivity, fertilizer application and nutrient use efficiency. In summary, it was suggested that best nutrient management practices, i.e. fertilizer recommendation applied based on soil testing or yield response, with strategies to match the nutrient input with realistic yield and demand, or provided with the 4R’s nutrient management (right time, right rate, right site and right fertilizer) should be adopted widely to improve the yield production and nutrient use efficiency.

## Introduction

Wheat (*Triticum aestivum* L.) is one of the most important cereal crops in China, playing a dominant role in maintaining food security and farmers’ income. With the increasing population and decreasing areas of arable land, improving the grain yield is the only way to meet the food demand. Grain yield increase is partly due to the use of improved varieties, and also partly attributable to better nutrient management [1–3]. Optimum nutrient management including fertilizer recommendation based on soil fertility and nutrient requirement could maintain sustainable yield, save fertilizer resources and improve fertilizer nutrient use efficiency. However, the fertilizers are usually not applied in balance, which results in low nutrient use efficiency.

The yield response (yield difference between NPK plots and corresponding nutrient omission plots), fertilizer contribution rate (FCR, the proportion of yield increase of N, P or K to the yield in NPK plots), partial factor productivity (PFP, kg grain yield produced per unit of N, P_2_O_5_ or K_2_O applied), agronomic efficiency (AE, kg grain yield increased per unit of N, P_2_O_5_ or K_2_O applied), and recovery efficiency (RE, the fraction of nutrient uptake in aboveground plant dry matter to the nutrient of fertilizer application) are frequently used in agronomic research to assess the efficiency of fertilizer application [4–7]. The indigenous nutrient supply is estimated by N, P and K uptake in aboveground plant dry matter in corresponding N, P and K omission plots. Excessive application and ignorance of the indigenous nutrient supply are usually the main reasons for low nutrient use efficiency. The nutrients not taken up by crops mostly remain in soil or are lost to the environment. Recent research showed that the indigenous N, P and K supply for wheat were high up to 123, 38 and 120 kg/ha, respectively [8]. The sources of indigenous nutrients include previous fertilizer application, deposition, irrigation, biological nitrogen fixation, and other sources except fertilization. Nitrogen not uptaken by crops can be lost by leaching, runoff or volatilization, and pose potential risk to the environment. Therefore, the nutrients in soil should be considered when making fertilizer recommendation to achieve the optimal nutrient management and high use efficiency.

In China, many different nutrient management practices were conducted to study their effects on nutrient use efficiency. While the investigation was usually conducted as an individual experiment at a certain location or conducted only in one year. It has been more than 30 years since the last national investigation of fertilizer use efficiency conducted in 1983 by the National Chemical Fertilizer Efficiency Study Network (NCFESN). Up to now, few systematic analyses have focused on the comparison and description of grain yield and nutrient use efficiency based on multiple years and multiple sites. Therefore, in this study, multiple datasets were selected from the International Plant Nutrition Institute (IPNI) China program database and published literatures during the years 2000 to 2011. The objectives were (1) to analyze the status and variation of grain yield and fertilizer application; and (2) to determine current nutrient use efficiency parameters including fertilizer contribution rate, partial factor productivity, agronomic efficiency and recovery efficiency.

## Materials and Methods

### Data sources

The grain yield, nutrients of N, P and K uptake in aboveground plant dry matter, harvest index (HI, kg grain per kg total aboveground dry matter), fertilizer application rates were collected from the regions of the Yellow and Huai River valley (YHRV), the middle and lower reaches of the Yangtze River (MLYZ), and Northwest China (NW). In YHRV, it mainly includes Hebei, Henan, Shandong, Shanxi, Shaanxi and Beijing provinces, and the main cropping system is a winter wheat-summer maize (*Zea mays* L.) rotation system, with wheat planted in mid-October after the harvest of maize. In MLYZ, it mainly includes Jiangsu, Hubei, Anhui and Hunan provinces, and the common practice is a rice (*Oryza sativa* L.)-wheat rotation with wheat sown in late October or early November after rice is harvested. In NW, it mainly includes Ningxia, Gansu and Xinjiang provinces, and usually spring wheat grown. Spring wheat is sown in March or April and harvested in July. These regions represented different growing environments in China. The number of the observations, climate and soil characters of experimental sites were listed in Chuan et al [8–9]. These treatments consisted of different management practices including the farmers’ practice (FP, fertilizer application followed the farmers’ practice), optimum practice (OPT, recommend fertilizer application followed soil testing or other science-based and reasonable guidance), nutrient omission plots, long-term field experiments, and treatments with different fertilizer rates.

In each experiment, three 1×1 m^2^ of grain and straw samples in the middle of each plot were usually collected to determine the yield. Subsamples of straw and grain were oven-dried at 60°C, and then digested with H_2_SO_4_–H_2_O_2_ [10]. The N, P and K concentrations were measured using the Kjeldahl method, vanadium molybdate yellow color method and flame spectrophotometer, respectively [10]. The total nutrient uptake of N, P and K were calculated from the nutrient concentration in subsamples multiplied by the plant dry weight.

### Calculations

To estimate the nutrient use efficiency of wheat, parameters of yield response, FCR, PFP, AE, and RE were calculated using the following equations [8, 11].

Yield response to N, P or K=grain yield in NPK plots −grain yield in N, P or K omission plots (1)

$$\text{FCR}=\frac{\text{yield response to N},\text{ P or K}}{\text{grain yield in NPK plots}}$$ (2)

$$\text{PFP for N},\text{ P or K}=\frac{\text{grain yield}}{\text{amount of N},{\text{ P}}_{2}{\text{O}}_{5}{\text{ or K}}_{2}\text{O fertilizer applied}}$$ (3)

$$\text{AE for N},\text{ P or K}=\frac{\left(\text{grain yield in NPK plots}-\text{grain yield in N},\text{ P or K omission plots}\right)}{\text{amount of N},{\text{ P}}_{2}{\text{O}}_{5}{\text{ or K}}_{2}\text{O fertilizer applied}}$$ (4)

$$\text{RE for N},\text{ P or K}=\frac{\left(\text{N},\text{ P or K uptake in aboveground plant in NPK plots}-\text{N},\text{ P or K uptake in aboveground plant in omission plots}\right)}{\text{amount of N},{\text{ P}}_{2}{\text{O}}_{5}{\text{ or K}}_{2}\text{O fertilizer applied}}$$ (5)

## Results

### Yield and fertilizer application

**Fig 1:**
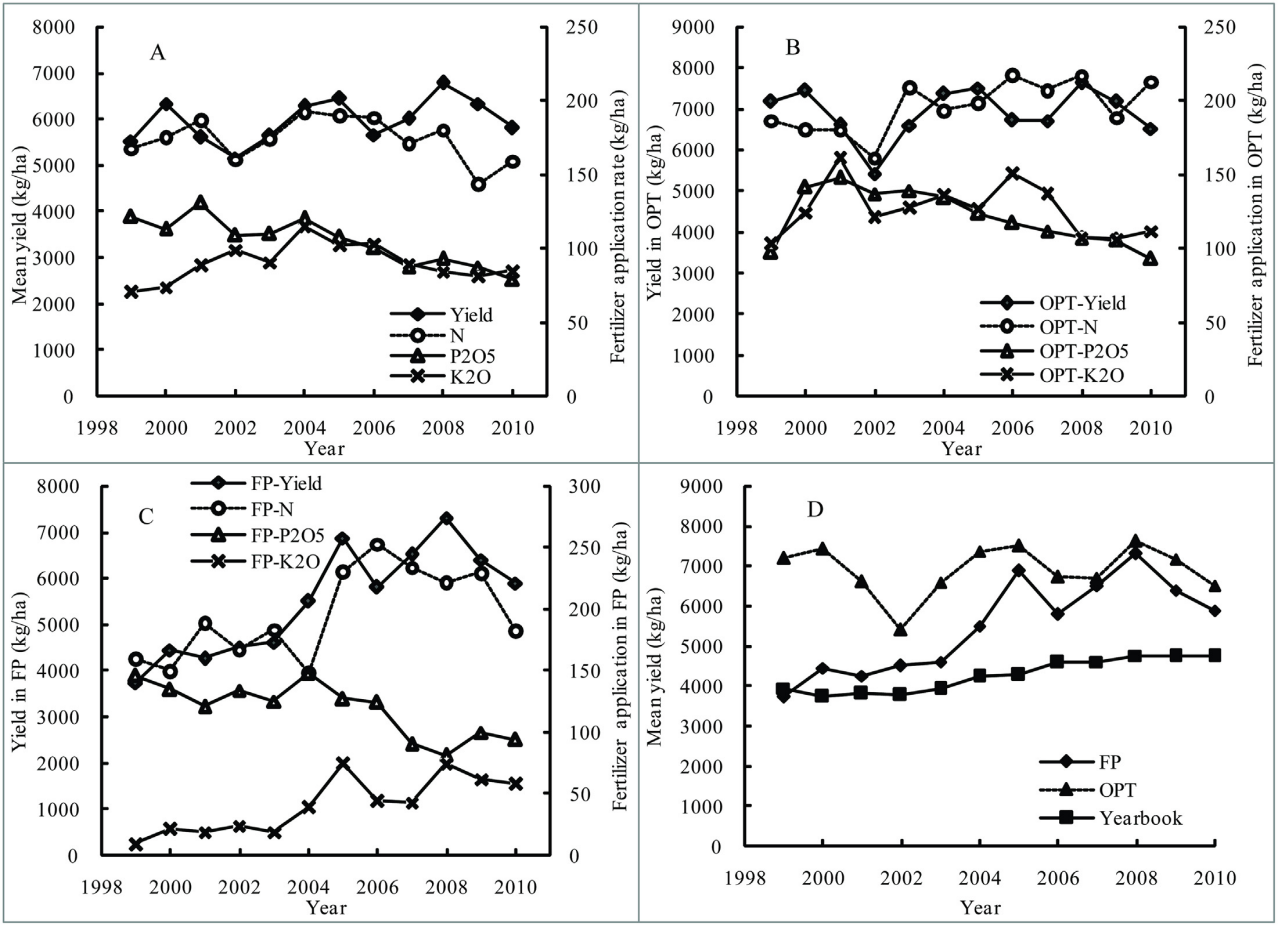
Changes of grain yield and fertilizer application for wheat in all treatments within different years (A), and for wheat only in OPT (B) and FP plots (C), respectively, and comparison of grain yield between OPT, FP plots and the yearbook (D) in different years.

The mean grain yield for wheat in China from 1999 to 2010 showed a general upward trend. The annual grain yield ranged from 5150 to 6785 kg/ha with an average of 5950 kg/ha for recent 12 years (Fig 1A). The average annual increase was 149 kg/ha, and the growth rate was 2.9%. The average rate of N fertilizer was 172 kg N/ha, ranging from 192 kg N/ha in 2004 to the rate of 144 kg N/ha in 2009, showing a trend of decrease in general. Before 2004, the N fertilizer application presented an uptrend with fluctuations, and then declined gradually until 2010. The average P_2_O_5_ fertilizer rate decreased from 131 kg/ha (2001) to 79 kg/ha (2010), with an average of 102 kg/ha. The K_2_O fertilizer application rate increased from 71 kg/ha in 1999 to 115 kg/ha in 2004, then slowly decreased and remained at around 80~90 kg/ha with an average of 91 kg/ha for the period of 1999–2010. Before 2004, the K_2_O fertilizer rate was much lower than P_2_O_5_; however, with the fertilizer application of K_2_O increase and P_2_O_5_ reduction, the rates of these two kinds of fertilizer were approximately at similar levels after 2004. The ratio of average N, P_2_O_5_ and K_2_O application rates in the period of 1999–2010 was 1:0.59:0.53. Compared with the ratio in the 1980s (N:P_2_O_5_:K_2_O = 1:0.63:0) [12], the decrease in N and P and increase in K fertilizer application balanced the nutrient supply and was the main reason for yield increase.

If only the values from OPT plots selected, the average yield ranged from 5420 kg/ha in 2002 to 7630 kg/ha in 2008 with an average of 6866 kg/ha, in general presenting an increasing trend with fluctuations (Fig 1B). The average N fertilizer application in OPT plots was 201 kg/ha (ranged from 161 kg/ha in 2002 to 217 kg/ha in 2008), showing a slight increase through the period of 1999–2010. However, the P_2_O_5_ fertilizer application rates decreased from 148 kg/ha in 2001 to 93 kg/ha in 2010, and K_2_O decreased from 161 kg/ha in 2001 to 106 kg/ha in 2009, with an average of 114 and 123 kg/ha, respectively.

A smaller datasets of FP treatments showed that the annual average yield ranged from 3746 kg/ha in 1999 to 7311 kg/ha in 2008 with an average of 5493 kg/ha, lower than that in OPT treatments (Fig 1C). The N, P_2_O_5_ and K_2_O fertilizers were applied in the ranges of 150~253 kg/ha, 82~148 kg/ha and 10~75 kg/ha, with an average of 196, 119 and 41 kg/ha, respectively. In general, N and K_2_O fertilizer showed a trend to slightly increase and P_2_O_5_ fertilizer showed to decline in the period of 1999–2010. Additionally, the yield in OPT plots was higher than that in FP plots, and the values in the yearbook were much lower than those of the OPT and FP plots (Fig 1D).

**Fig 2:**
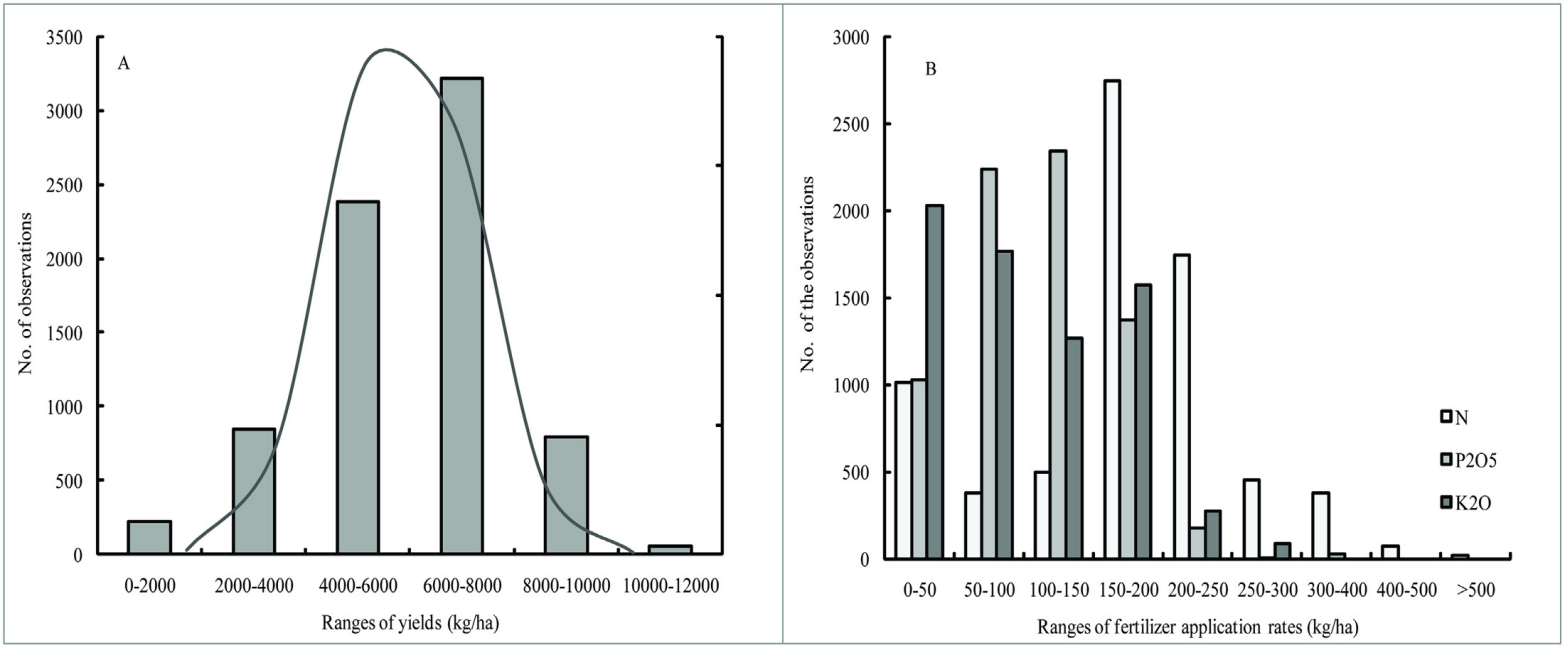
The distribution of different ranges of grain yield (A) and N, P_2_O_5_ and K_2_O fertilizer application (B).

The grain yield and N, P_2_O_5_ and K_2_O fertilizer application rates showed a large variation in recent years, especially the yield, ranging from 280 kg/ha to 12000 kg/ha. However, the yield presented a normal distribution characteristic (Fig 2A). There were 31.7% and 42.8% of the data were distributed in 4000~6000 kg/ha and 6000~8000 kg/ha, with low frequency distribution in the extremely low and high yield ranges. The distribution of N, P_2_O_5_ and K_2_O fertilizer application indicated that 37.6% of N, 32.6% of P_2_O_5_ and 29.0% of K_2_O fertilizer applied in the ranges of 150~200, 100~150 and 0~50 kg/ha, respectively (Fig 2B). The N fertilizer varied from 0 to 600 kg/ha, presenting larger ranges of variation. Approximately, up to one third of the K_2_O fertilizer application rates were lower than 50 kg/ha, which could result in K depletion in the soils that did not have adequate K stock.

**Fig 3:**
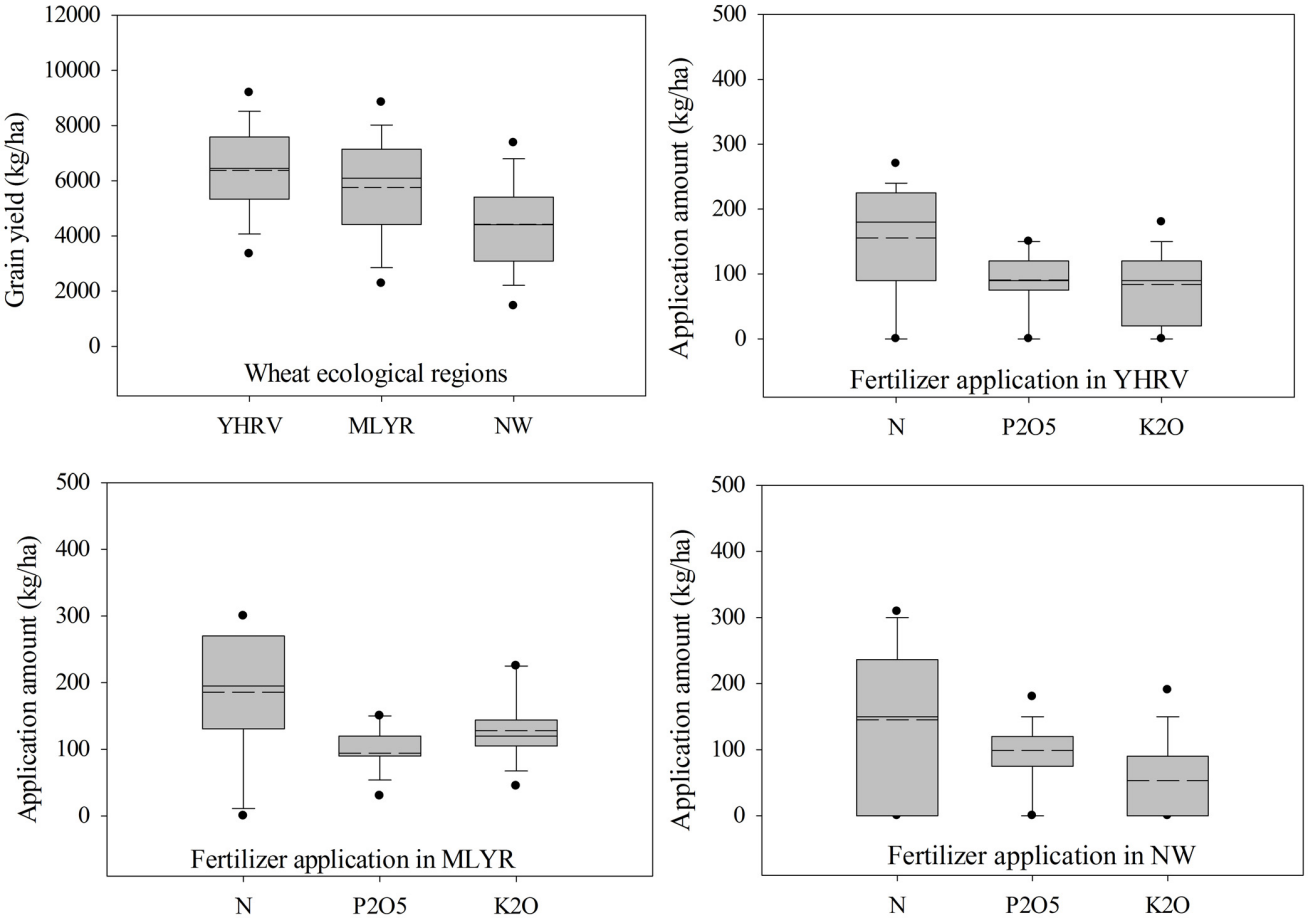
The grain yield and fertilizer application in the Yellow and Huai River valley (YHRV) the middle and lower reaches of the Yangtze River (MLYR) and Northwest China (NW) China.

Different wheat ecological regions differ greatly in wheat productivity and fertilizer application. The results showed that the average grain yield in YHRV, MLYR and NW regions were 6378.5, 5756.2 and 4426.1 kg/ha, respectively, indicating that the wheat yield in YHRV was higher than MLYR and NW region (Fig 3). The average fertilizer N, P_2_O_5_ and K_2_O in YHRV were 155.7, 90.7, 83.9 kg/ha, and in MLYR were 185.8, 94.3, 128.0 kg/ha, and in NW were 145.3, 99.1, 53.1 kg/ha, respectively, showing the N and K_2_O fertilizer application in MLYR were higher than YHRV and NW region, and the P_2_O_5_ fertilizer application were similar in these three regions.

### Yield responses and fertilizer contribution rates

**Fig 4:**
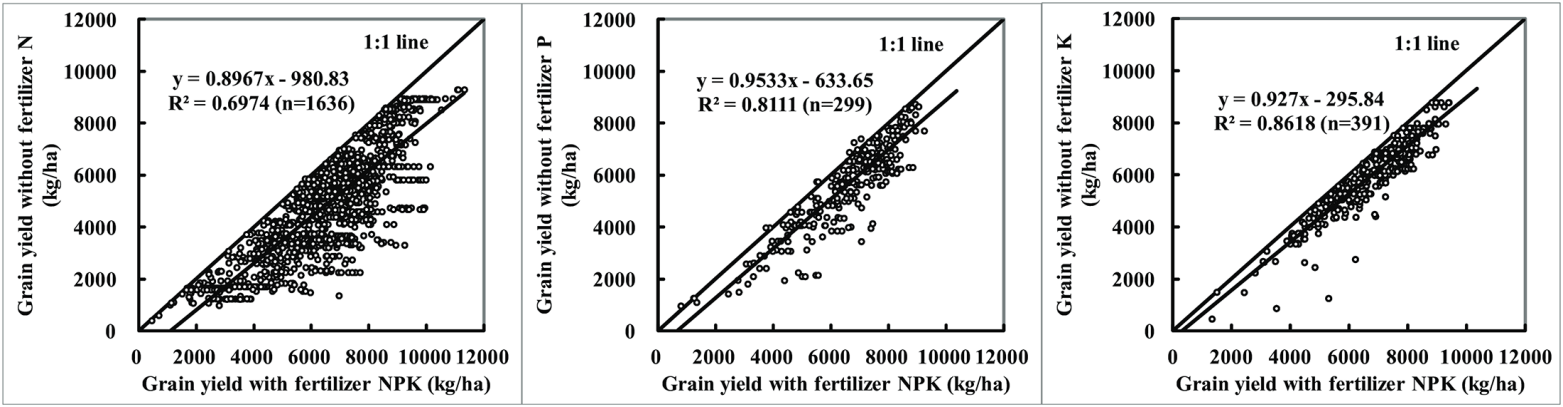
Relationship in grain yield between NPK plots and N, P or K omission plots.

Yield between the NPK plots and corresponding omission plots showed a highly significant positive linear correlation (*p*<0.01) (Fig 4). The yield in NPK plots increased with increasing yield in corresponding N, P and K omission plots, and the correlation coefficients were 0.84, 0.90 and 0.93 (*p*<0.01), respectively, indicating that the nutrient in the omission plots supplied by the soil indigenous fertility had a significant effect on the yield production. Additionally, the lower correlation coefficient, the higher important role of this omitted nutrient in the yield increase. If the simulated curve was closer to the 1:1 line, this omitted nutrient would have lower influence on the yield production. Therefore, the result showed that the influence of omitted N nutrient on the yield formation was higher than P, and then K on the yield formation.

**Fig 5:**
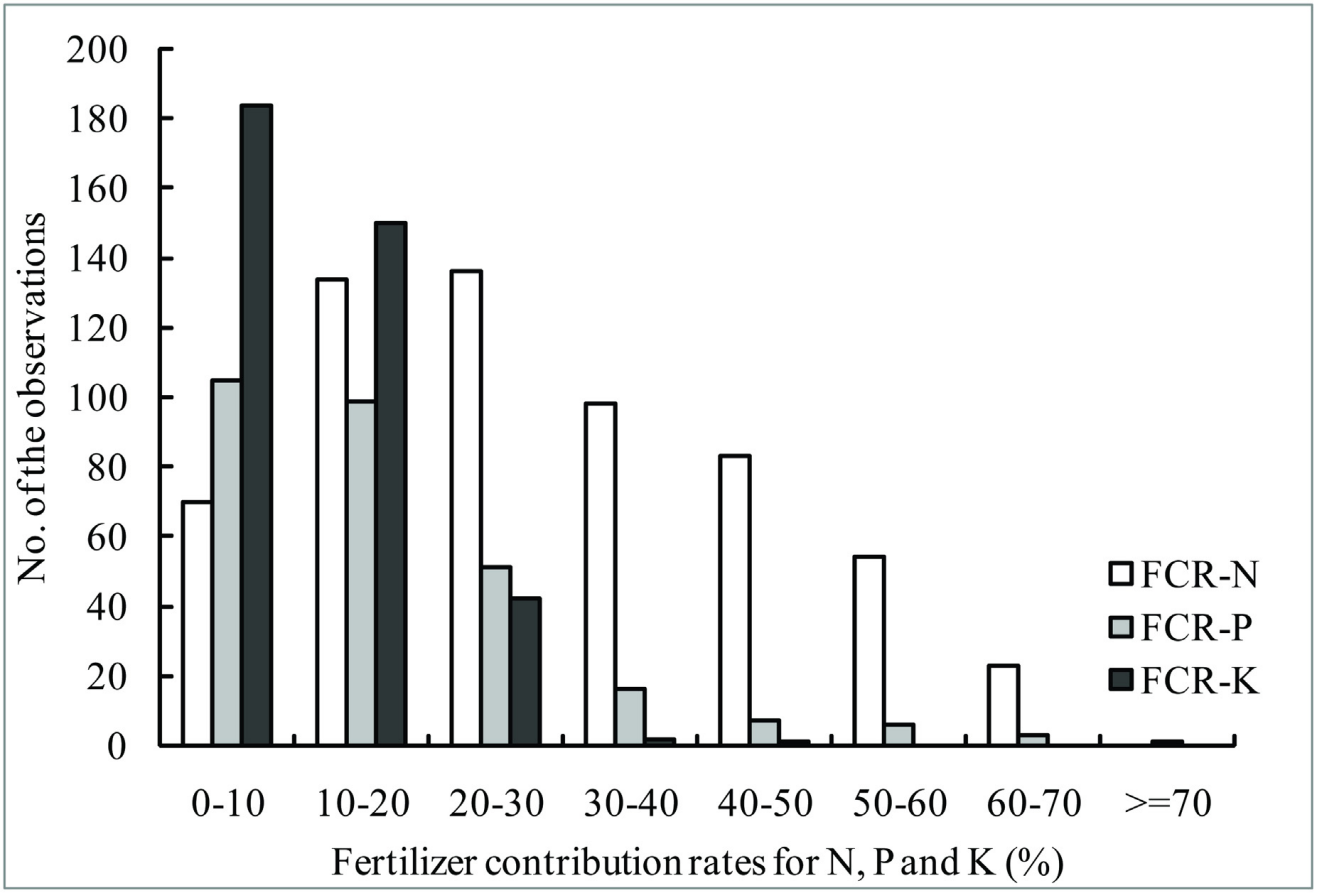
The distribution of fertilizer contribution rates of N, P and K (FCR-N, FCR-P and FCR-K).

The grain yield in N omission plots (PK) ranged from 1077 kg/ha to 9292 kg/ha with an average of 4628 kg/ha (Table 1). Compared with the yield in OPT treatments (best nutrient management practices), the mean yield response to N was 1807 kg/ha. The yield in P and K omission plots ranged from 520 to 8735 kg/ha and 1490 to 8784 kg/ha, and the yield responses to P and K were 955 and 771 kg/ha, respectively. There were 56.9% of the value of fertilizer contribution rates for N (FCR-N) lower than 30%, and 36.5% and 34.4% of the value of fertilizer contribution rates for P (FCR-P) in the ranges of 0~10% and 10~20%, and 48.6% and 39.6% of the value of fertilizer contribution rates for K (FCR-K) fell in the ranges of 0~10% and 10%~20%, respectively, which were mostly lower than FCR-N (Fig 5). The average value of FCR-N, FCR-P and FCR-K were 29.3%, 16.3% and 11.3%, respectively. Therefore, N was the first nutrient limiting factor, followed by P, and then K.

**Table 1:** The distribution of grain yield, yield response and fertilizer contribution rate for wheat.

Treatments	Unit	n^a^	Mean	SD^b^	Minimum	25%Q^c^	Median	75%Q	Maximum
**PK**	kg/ha	601	4628	2029	1077	3218	4689	5929	9292
**NPK**	kg/ha	601	6435	2094	1203	4799	6900	7959	11312
**Yield response to N**	kg/ha	601	1807	1103	32	928	1652	2495	5336
**N contribution rate**	%	601	29.3	16.6	0.5	16.1	27.6	41.6	80.4
**NK**	kg/ha	288	5735	1733	520	4925	6118	6855	8735
**NPK**	kg/ha	288	6690	1648	1347	6008	7200	7926	9234
**Yield response to P**	kg/ha	288	955	782	8	506	782	1300	3964
**P contribution rate**	%	288	16.3	13.1	0.2	7.0	13.4	21.4	88.4
**NP**	kg/ha	380	6095	1275	1490	5280	6242	6862	8784
**NPK**	kg/ha	380	6866	1338	1501	6040	7194	7842	9401
**Yield response to K**	kg/ha	380	771	464	1	419	677	1076	2493
**K contribution rate**	%	380	11.3	6.6	0.1	6.5	10.5	15.6	41.1

^a^ n, number of observations.

^b^ SD, standard deviation.

^c^ Q, quartile.

### Fertilizer partial factor productivity

**Fig 6:**
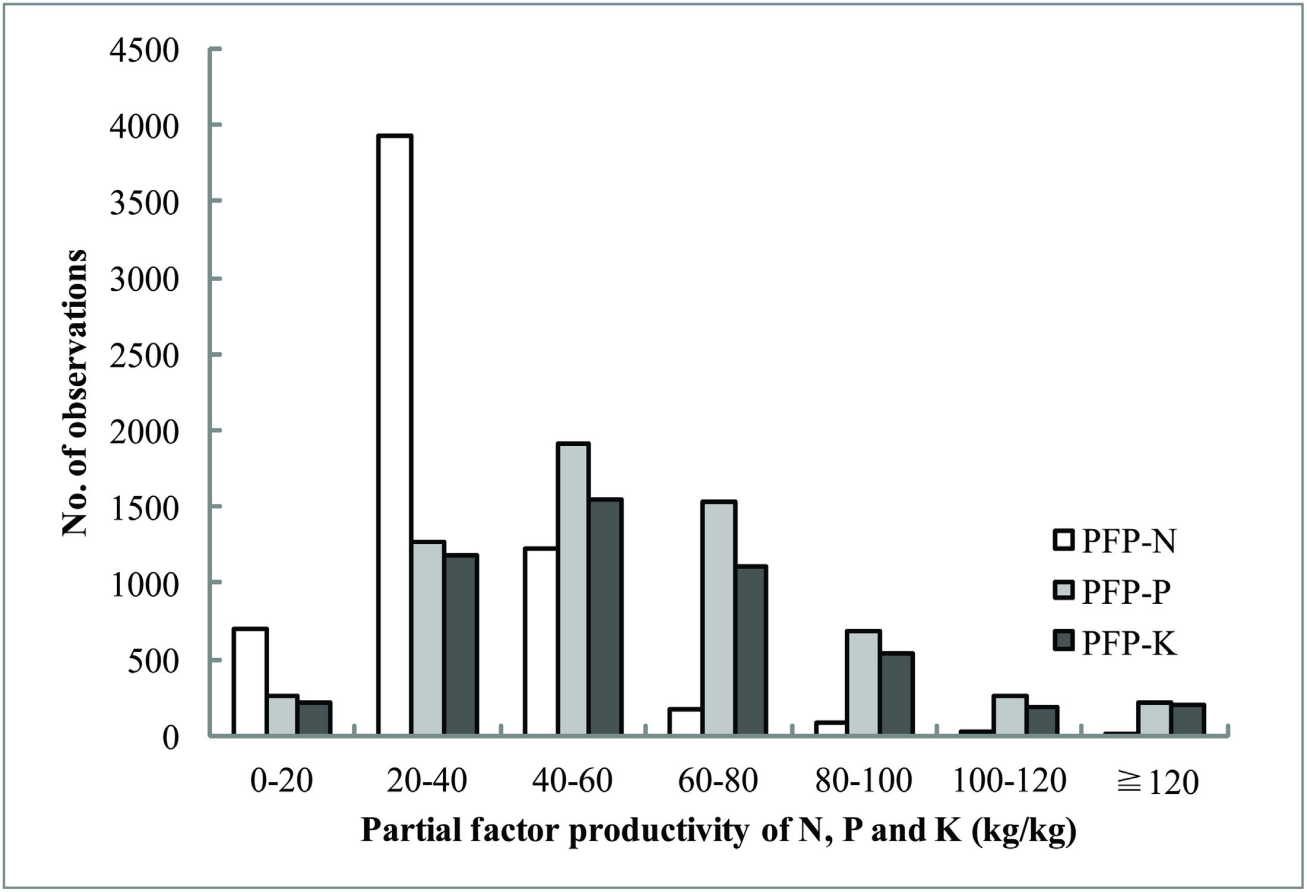
The distribution of partial factor productivity of N, P and K fertilizer (PFP-N, PFP-P and PFP-K).

Fertilizer partial factor productivity was usually used to evaluate the fertilizer use efficiency in a large scale since it was easily obtained. The results showed that the average of PFP for N (PFP-N), P (PFP-P) and K (PFP-K) were 34.2, 60.3 and 58.5 kg/kg, respectively. Up to 63.8% of the PFP-N observations were in the range of 20~40 kg/kg, and 31.2% of PFP-P and 31.1% of PFP-K distributed at 40~60 kg/kg, respectively (Fig 6).

**Fig 7:**
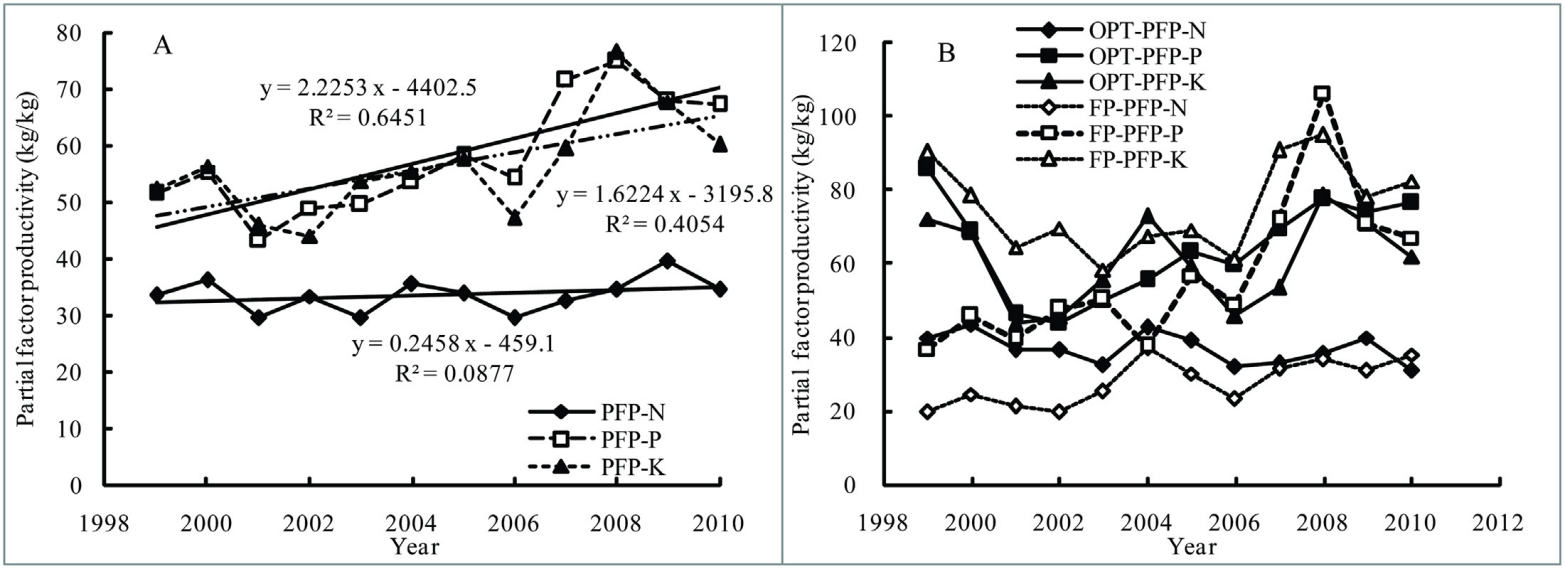
Changes of partial factor productivity of N, P and K fertilizer (PFP-N, PFP-P and PFP-K) for all treatments (A) and for only OPT and FP plots (B) in different years, respectively.

The PFP-N values were mostly lower than the values or PFP-P and PFP-K, and the annual average values ranged from 29.5 kg/kg to 39.6 kg/kg, with slight variations in the period of 1999 to 2010 (Fig 7A). The PFP-P and PFP-K showed an increasing trend, especially after 2006. The PFP-P increased from 43.4 kg/kg (2001) to 74.9 kg/kg (2008) with the annual growth rate of 10.4%. The PFP-K grew from 44.1 kg/kg (2002) to 76.5 kg/kg (2008) with the annual increase of 12.2%. This was closely related to the yield improvement and P and K fertilizer rates reduction.

The PFP-N in OPT plots (ranged from 31.1 kg/kg to 43.5 kg/kg) was mostly higher than that in FP plots (ranged from 23.3 kg/kg to 37.1 kg/kg) (Fig 7B). However, the PFP-P in OPT plot before 2007 was higher than that in FP plots and then was lower due to the gradual decrease of P application rate. Because of lower K applied in FP plots, the PFP-K was mostly higher than that in OPT plots.

### Agronomic efficiency and recovery efficiency

**Fig 8:**
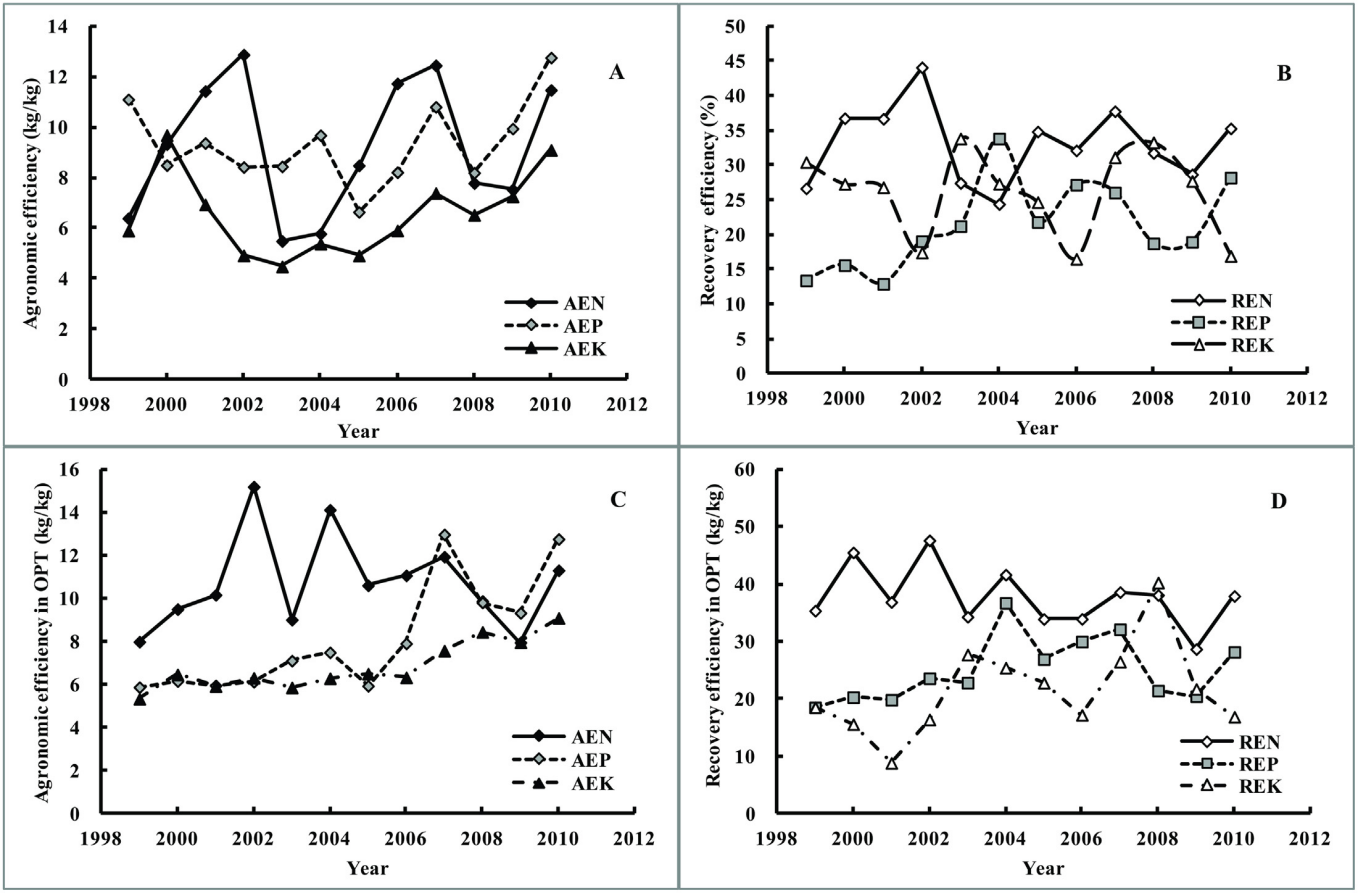
Agronomic efficiency (A) and recovery efficiency (B) of N, P and K for all treatments in different years, and Agronomic efficiency (C) and recovery efficiency (D) of N, P and K for OPT plots in different years. AEN, AEP and AEK mean the agronomic efficiency of N, P and K; REN, REP and REK mean the fertilizer recovery efficiency of N, P and K.

Both agronomic efficiency and recovery efficiency could be used to characterize the nutrient effects [6, 13]. The average agronomic efficiency of N (AEN), P (AEP) and K (AEK) were 9.4, 10.2 and 6.5 kg/kg (Table 2), showing a fluctuating curve. Meanwhile, the amplitude of fluctuation for AEN was bigger than that for AEP and AEK, and AEK values were generally lower than AEN and AEP (Fig 8A). After 2005, AEN, AEP and AEK presented the similar trend of variability, but AEK was still lower than AEP. While after 2008, AEP was higher than AEN.

**Table 2:** Characteristics of agronomic efficiency and recovery efficiency of N, P and K for wheat.

Parameters	unit	n^a^	Mean	SD^b^	Minimum	25%Q^c^	Median	75%Q	Maximum
**AEN**^d^	kg/kg	1607	9.4	6.5	0.0	4.4	8.0	13.0	37.8
**AEP**	kg/kg	288	10.2	6.7	0.1	5.3	9.2	13.8	39.7
**AEK**	kg/kg	380	6.5	3.8	0.0	3.6	6.0	8.4	19.1
**REN**^e^	%	1549	33.1	17.2	0.4	19.8	31.3	44.2	88.2
**REP**	%	217	24.3	14.7	0.2	12.0	24.0	36.5	61.2
**REK**	%	381	28.4	20.0	0.3	14.4	23.7	36.0	93.1

^a^ n, number of observations.

^b^ SD, standard deviation.

^c^ Q, quartile.

^d^ AEN, AEP, and AEK mean the agronomic efficiency of N, P and K.

^e^ REN, REP, and REK mean the fertilizer recovery efficiency of N, P and K.

Great variation was observed for RE. The recovery efficiency of N (REN), P (REP), and K (REK) ranged from 0.4% to 88.2%, 0.2% to 76.5% and 0.3% to 93.1%, with an average of 33.1%, 24.3% and 28.4% in the period of 1999 to 2010, respectively (Table 2). Before 2002, the REN was higher, then it declined sharply to 24.4% in 2004, and afterwards it gradually changed to increase with fluctuations (Fig 8B). The REP reached the peak in 2004, and then varied with fluctuations. The REK showed a great variation in the period of 1999–2010, but in general it had no significant improvement.

The AEN in OPT plots ranged from 8.0 to 15.2 kg/kg, varied with the years but to lesser extents compared to the variation in AEN in all plots (Fig 8C). However, the AEP (ranged from 6.0 to 13.0 kg/kg) and AEK (ranged from 5.4 to 9.1 kg/kg) both showed a large increase within the observed years. The AEN and AEP were mostly higher than AEK. Before 2007, the AEN was much higher than AEP and AEK, and later AEP became a little higher than AEN.

The RE of N, P and K in OPT plots were shown in Fig 8D. The results showed that the REN, REP and REK ranged from 28.7% to 47.6%, 20.5% to 36.8% and 8.9% to 40.3%, respectively, while presenting a large variation from 1999 to 2010.

The AE and RE of N, P and K from FP plots were also analyzed (data were not shown). Considering observations for P and K were few, and then only N use efficiency was discussed. The AEN for FP ranged from 3.4 to 11.1 kg/kg and REN ranged from 13.6% to 28.5%, which were both lower than that in OPT plots.

### Nutrient use efficiency in different ecological regions

Nutrient use efficiency was also analyzed in different ecological regions (Table 3). The results showed that the FCR-N, FCR-P and FCR-K in YHRV were 25.2%, 16.3% and 11.6%. FCR-N was lower than that in MLYR (43.7%), and FCR-P and FCR-K were both higher than that in MLYR and NW regions. The PFP-N in YHRV and MLYR regions were 37.0 and 32.9 kg/kg, which were both higher than that in NW region, and PFP-P and PFP-K in YHRV region were 72.3 and 71.1 kg/kg, also higher than that in MLYR (62.9, 48.0 kg/kg) and NW regions (33.8, 41.1 kg/kg). The AEN in the above three regions were 8.0, 13.4 and 6.7 kg/kg, showing the AEN in MLYR region was highest. While AEP and AEK in these three regions were all lower than 10.0 kg/kg. The REN, REP and REK in MLYR region (41.5%, 28.3% and 40.6%) were all higher than that in YHRV (29.9%, 24.6% and 27.5%) and NW region (22.0%, 7.1% and 14.7%), also representing the lower nutrient use efficiency in China.

**Table 3:** The fertilizer contribution rate (FCR), partial factor productivity (PFP), agronomic efficiency (AE) and recovery efficiency (RE) of N, P and K for wheat in different ecological regions.

Nutrient use efficiency	YHRV	MLYR	NW
**FCR-N**	25.2	43.7	22.0
**FCR-P**	16.3	10.7	13.4
**FCR-K**	11.6	7.9	7.2
**PFP-N**	37.0	32.9	23.1
**PFP-P**	72.3	62.9	33.8
**PFP-K**	71.1	48.0	41.1
**AEN**	8.0	13.4	6.7
**AEP**	9.8	9.1	6.1
**AEK**	6.5	5.8	3.8
**REN**	29.9	41.5	22.0
**REP**	24.6	28.3	7.1
**REK**	27.5	40.6	14.7

## Discussions

### Yield and fertilizer application

The average yield and fertilizer application rates calculated from the multi-year and multi-point datasets showed general characteristics under different nutrient management practices, rather than only farmers’ practices. Due to the large amount of phosphate and compound fertilizer production, the P fertilizer application rates remained very high in the early 2000s [14]. While with the development of soil testing technology, farmers realized that scientific fertilization could improve the yield and gradually reduced the amount of P fertilizer after 2004. During the period of 1999–2004, because of the advocating of K fertilizer application, a group of potash companies gradually evolved; and also because farmers changed from urea to compound fertilizer, the K fertilizer application rates gradually increased. However, K fertilizer rates slightly decreased after 2004 in order to adjust the balanced ratio of N, P and K fertilizers with the soil testing implemented in large areas of China. In general, the popularization of scientific fertilization and the raising awareness of farmers’ scientific cultivation both had the greatest influence on the fertilizer application.

In this study, the average yield for wheat was 5950 kg/ha. A previous study showed that the yield potential in the Huang-Huai-Hai Plain was 10500 kg/ha [15]. The high yield observations in multi-year and multi-point datasets indicated that the breeding and cultivation practices on high yield varieties had been gradually matured, while the yield potential had not been fully realized in most areas of China. There were still 45.9% of the observations lower than 6000 kg/ha, suggesting that the yield had large scopes to improve, especially in low productivity areas. It is well known that yield is the combined performance of fertilizer, soil, light, temperature and water. For a specific area, soil, light and temperature are usually out of control, the best nutrient management for crops under full irrigation condition, combined with the 4R technology (right time, right fertilizer source, right site, and right rate), will make great contribution for the yield increase [16].

### Nutrient use efficiency

Based on results of 782 field trials, Zhu and Wen [17] summarized that the REN of the main cereal crops was in the range of 28%~41% with an average of 35%, and REP ranged from 6% to 26%, and REK was about 50%, higher than REN and REP. In 1998, Zhu [18] stated further that the REN, REP and REK for the cereals were in the ranges of 30%~35%, 15%~20% and 35%~50%, respectively. Liu et al. [19] also reported that the REN, REP and REK for wheat were 45%, 22% and 47% in 1985–1995, respectively. Zhang et al. [20] calculated the REN, REP and REK from 273, 150 and 165 wheat samples in 2001–2005 were 28.2%, 10.7% and 30.3%. In this study, the REN, REP and REK for wheat were found to be 33.1%, 24.3% and 28.4% in the period of 1999 to 2010, respectively. From a historical perspective, the REN in the 2000s was lower than that in the 1980s and 1990s. This was mainly because the amount of N fertilizer application in previous years was lower than recent years. Another reason was that farmers applied more organic fertilizers in previous years, but with the increase in of chemical fertilizer application in recent years, the recovery efficiency gradually decreased. The REP varied greatly within the different periods, and REK decreased over the years with the increased K fertilizer application.

Compared with the AE data from NCFESN by the Ministry of Agriculture in the period of 1958–1962 and 1981–1983 [21] (Table 4), AEN declined mainly due to the excessive N fertilizer application in many observations; AEP was slightly higher in the period of 1999–2010, and AEK values were all lower than AEN and AEP, but improved mainly due to the depletion of available K in the soil. It is important to note that the average AEP in the period of 1999–2010 was higher than AEN and AEK, which was very different from the previous studies. The nutrient use efficiency had a large variation in the different experiments in this study. The lower AE values were usually from the higher rate fertilizer application trials with relatively high soil fertility; the higher AE values were usually from the optimum nutrient management practice; and the AE values in OPT plots were highest among all the datasets collected. The OPT treatments applied the best nutrient management practices, i.e. fertilizer recommendation applied was based on soil testing or yield response, with strategies to match the nutrient input with realistic yield and demand, or provided with the 4R’s nutrient management (right time, right rate, right site and right fertilizer). Therefore, all above good nutrient management practices should be adopted widely to improve the nutrient use efficiency.

**Table 4:** The agronomic efficiency of N, P and K (AEN, AEP and AEK) for wheat in different periods in China.

Year	Fertilizer N application (kg/ha)	AEN (kg/kg)	Fertilizer P_2_O_5_ application (kg/ha)	AEP (kg/kg)	Fertilizer K_2_O application (kg/ha)	AEK (kg/kg)
**1958–1962**^a^	45–60	10–15	45–60	5–10	45–60	0
**1981–1983**	117 (1462)^b^	10.0	81.0 (1851)	8.1	85.5 (678)	2.1
**2000–2005**	169 (273)	8.0	114 (150)	7.3	110 (165)	5.3
**1999-2010(OPT)**	206 (383)	9.9	103 (154)	10.6	112 (160)	7.5
**1999–2010**	172 (1607)	9.4	102 (288)	10.2	91 (380)	6.5

^a^, the data for 1958–1962, 1981–1983 and 2000–2005 were quoted by Wang et al. (2008).

^b^, the value in the parentheses was the number of the observations.

Fertilizer nutrients that were not taken up by the crop may pose negative effects to the environment. This is especially the case for N, which can be lost through leaching, erosion, denitrification or volatilization. A review of worldwide data on the nutrients use efficiency for cereal crops from researcher-managed experimental plots reported that the average REN was 57% for wheat [22]. Dobermann [6] suggested that the AE and RE for N should range from 10 to 30 kg/kg and 30% to 50%, and also suggested that with lower levels of N fertilizer application and best nutrient management, AE should be greater than 25 kg/kg, and RE could achieve 50%~80% [6]. The fertilizer use efficiencies in China were low, mainly due to farmers’ pursuit of higher yield targets resulting in undisciplined fertilizer application, inappropriate fertilizer sources and inefficient management practices.

Science-based and reasonable fertilization guidance is essential for crop production and critical for improving nutrient use efficiency. Soil testing could increase crop yield and improve nutrient use efficiency through the use of science-based nutrient recommendations [11]. Another method is based on the crop yield response, i.e., the optimum fertilizer rate is estimated by the yield difference between the attainable yield and the yield in corresponding nutrient omission plots, and then adjusted by the agronomic efficiencies [23]. A computer based system called *Nutrient Expert for Wheat* developed by the International Plant Nutrition Institute (IPNI) has been implemented in North Central China. It could help maintain crop production, maximize the use of soil indigenous nutrient supply, and thus improve the nutrient use efficiency [23, 8]. If the scientific technologies of fertilization and cultivation adopted, the nutrient use efficiency would be improved for crop production.

## Conclusions

The average grain yield of wheat calculated from the field trial results across multiple sites over 2000–2011 was 5950 kg/ha; the N, P_2_O_5_ and K_2_O application rates were 172, 102 and 91 kg/ha, respectively. The rates of N and P_2_O_5_ application decreased while K_2_O application increased during this period, which resulted in more balanced nutrient supply and was the main reason for yield increase.

The PFP of N, P and K were in the ranges of 29.5~39.6, 43.4~74.9 and 44.1~76.5 kg/kg, respectively. The PFP-N was mostly lower than PFP-P and PFP-K. In addition, PFP-N did not change significantly from 2000 to 2010, while both PFP-P and PFP-K showed an increasing trend over this period. The mean AEN, AEP and AEK were 9.4, 10.2 and 6.5 kg/kg, respectively, showing a fluctuating curve. Meanwhile, the amplitude of fluctuation for AEN was bigger than AEP and AEK, and values of AEK were generally lower than AEN and AEP. The mean REN, REP and REK were 33.1%, 24.3%, and 28.4%, respectively. The result was also revealed that different wheat ecological regions differ greatly in wheat productivity, fertilizer application and nutrient use efficiency. It was suggested that if farmers adopt the advanced technologies of fertilization and cultivation, i.e. fertilizer recommendation applied based on soil testing or yield response, with strategies to match the nutrient input with realistic yield and demand, or provided with the 4R’s nutrient management (right time, right rate, right site and right fertilizer), the nutrient use efficiency would be improved in wheat production.

## Supporting Information

S1 Fig Changes of grain yield and fertilizer application for wheat in all treatments within different years (A), and for wheat only in OPT (B) and FP plots (C), respectively, and comparison of grain yield between OPT, FP plots and the yearbook (D) in different years. (XLSX)

S2 Fig The distribution of different ranges of grain yield (A) and N, P_2_O_5_ and K_2_O fertilizer application (B). (XLSX)

S3 Fig The grain yield and fertilizer application in the Yellow and Huai River valley (YHRV) the middle and lower reaches of the Yangtze River (MLYR) and Northwest China (NW) China. (XLSX)

S4 Fig Relationship in grain yield between NPK plots and N, P or K omission plots. (XLSX)

S5 Fig The distribution of fertilizer contribution rates of N, P and K (FCR-N, FCR-P and FCR-K). (XLSX)

S6 Fig The distribution of partial factor productivity of N, P and K fertilizer (PFP-N, PFP-P and PFP-K). (XLSX)

S7 Fig Changes of partial factor productivity of N, P and K fertilizer (PFP-N, PFP-P and PFP-K) for all treatments (A) and for only OPT and FP plots (B) in different years, respectively. (XLSX)

S8 Fig Agronomic efficiency (A) and recovery efficiency (B) of N, P and K for all treatments in different years, and Agronomic efficiency (C) and recovery efficiency (D) of N, P and K for OPT plots in different years. (XLSX)

S1 Table The distribution of grain yield, yield response and fertilizer contribution rate for wheat. (XLSX)

S2 Table Characteristics of agronomic efficiency and recovery efficiency of N, P and K for wheat. (XLSX)

S3 Table The fertilizer contribution rate (FCR), partial factor productivity (PFP), agronomic efficiency (AE) and recovery efficiency (RE) of N, P and K for wheat in different ecological regions. (XLSX)

S4 Table The agronomic efficiency of N, P and K (AEN, AEP and AEK) for wheat in different periods in China. (XLSX)
